# Measurement of Indocyanine Green as a Predictor of Liver Failure After Hepatic Resection, Contributing to Risk Stratification in Personalized Medicine

**DOI:** 10.3390/jpm15100488

**Published:** 2025-10-13

**Authors:** Víctor Baladrón González, David Padilla Valverde, María del Carmen Gasco García, Pedro Juan Villarejo Campos, María Jesús Pardo Mora, Natalia Bejarano Ramírez, Omar Montenegro Herrera, Patricia Faba Martín, Rubén Villazala González, Francisco Javier Redondo Calvo

**Affiliations:** 1 Department of Anesthesiology and Critical Care Medicine, University General Hospital, 13004 Ciudad Real, Spain; vbaladron@sescam.jccm.es (V.B.G.); ommontenegro@sescam.jccm.es (O.M.H.); pfaba@sescam.jccm.es (P.F.M.); rvillazala@sescam.jccm.es (R.V.G.); 2 Faculty of Medicine, Universidad de Castilla-La Mancha, 13071 Ciudad Real, Spain; davidp@sescam.jccm.es; 3 Department of Surgery, University General Hospital, 13005 Ciudad Real, Spain; 4 Department of Anesthesiology, School of Dentistry, Complutense University, 28040 Madrid, Spain; mcgasco@med.ucm.es; 5 Department of Surgery, University Hospital Fundación Jiménez Díaz, 28040 Madrid, Spain; villarejocampos@yahoo.es; 6 Healthcare Center Ciudad Real 2, 13001 Ciudad Real, Spain; mjpardom@sescam.jccm.es; 7 Department of Pediatrics, University General Hospital, 13004 Ciudad Real, Spain; 8 Translational Research Group, GAI of Ciudad Real, Research Institute of Castilla-La Mancha (IDISCAM), 13004 Ciudad Real, Spain

**Keywords:** indocyanine green clearance rate, liver failure, pulse densitometry, personalized medicine

## Abstract

**Background**: Most of the advances in liver surgery have been achieved in the last few decades. The development of new diagnostic and therapeutic techniques has aided diagnosis and has facilitated more efficient and personalized resections for liver disorders. The estimation of the hepatic reserve has gained great importance because it marks the limit for more aggressive liver resections. It was hypothesized that determination of hepatic reserve by measuring plasma clearance of indocyanine green—following hepatic parenchymal liver resection—could provide earlier and more accurate knowledge of hepatic reserve and thus allow for more personalized therapy. **Methods**: A prospective observational post-authorization study was performed. **Results**: Applying ROC curves and the area under the curve (AUC) for the evaluation of the different tests as predictors of liver failure, favorable data were obtained in relation to bilirubin (AUC = 0.922) and prothrombin time (AUC = 1), and for postoperative PDR (AUC = 0.879) and GOT (AUC = 0.857), but not for preoperative PDR (AUC = 0.667) or GPT (AUC = 0.6). **Conclusions**: The gold standard for predicting early liver failure (the 50:50 criterion at on postoperative day 5) has a very good relationship with the plasma clearance rate of indocyanine green on postoperative day 1 and therefore has the potential to support earlier and more personalized therapeutic interventions, pending further validation.

## 1. Introduction

Most of the progress in liver surgery has been achieved in recent decades. The development of new diagnostic and therapeutic techniques has aided diagnosis and allowed the more efficient use of resections, ablations and other treatments of liver disorders. Major hepatectomy has reduced morbidity to less than 30% and mortality to below 5%. This can be performed in patients for whom resection is often the only curative option [1,2,3]. In order to perform a greater number of liver resections, new diagnostic techniques that facilitate the identification of the liver reserve have been introduced, since liver resection is limited by the need to preserve enough healthy tissue for the liver to continue to perform its functions. In this context, personalized medicine offers a framework for tailoring surgical strategies to the individual patient’s hepatic function. Resection of a surplus of parenchyma can lead to liver failure and death of the patient in a short period of time [4].

Liver failure is a rare but life-threatening condition. Liver disorders affect a large number of other organs. Patients with hepatic insufficiency will have longer hospital stays and a greater consumption of resources, with a negative impact on their survival. In order to predict post-operative liver failure in resective liver surgery, different clinical, radiological and analytical methods have been used with the intention of making the surgery safer and increasing the indications or liver resection [5,6].

The estimation of the liver reserve has become very important in recent years, because it marks the limit for the most aggressive liver resections. The ideal test does not yet exist, because the great complexity of liver functions makes it difficult to find a test that is capable of quantifying all of them. Conventional tests only quantify the activity or concentration of a substance in the blood, and this depends on the relationship between its volume of distribution and the difference between its rates of production and elimination. To be useful, the substance should only be produced and/or disposed of in the liver. Quantitative tests can provide information on hepatic function by measuring the elimination of a substance previously introduced into the patient [7].

Indocyanine green (ICG) is a water-soluble dye with a spectral absorption peak of 800 nanometres (nm). It was introduced into clinical research by Fox and Brooker in 1956. It has been used for more than half a century to calculate cardiac output, regional blood flow and hepato-splanchnic flow [8]. When administered intravenously, it binds to plasma proteins and is eliminated unaltered in bile, without experiencing enterohepatic recirculation, thus allowing estimation of the function of hepatocytes and hepato-splanchnic flow. There is currently a non-invasive liver function monitor (LiMON^®^, PULSION Medical Systems, Munich, Germany) that has a good correlation with invasive systems [9,10]. The non-invasive monitor has allowed more widespread use of indocyanine green, as its elimination can be measured at the patient’s bedside, facilitating its integration into personalized medicine strategies.

Indocyanine green is contraindicated for people with hypersensitivity to it or to iodine, and for patients with hyperthyroidism, thyroid adenomas, and focal or diffuse thyroid autonomies. It is not indicated for neonates or preterm infants who may require exchange transfusion. ICG should be used with caution in patients with renal failure and in those who take beta-blockers. The main side effects described are nausea, hives, rash, tachycardia and anaphylactoid reactions such as laryngospasm, bronchospasm and hypotension.

The non-invasive measurement of indocyanine green is based on pulse spectrophotometry. By measuring the ICG clearance (ICG-PDR) by spectrophotometry, a graph representing the dye removal can be obtained. For the first two minutes of ICG removal, the elimination is exponential. The elimination slope is calculated by linear regression of a semilogarithmic trace between 2.5 and 5.5 min of the mean transit time, or the average time that is required for the substance to be distributed throughout the circulatory system. An extrapolation is then made to calculate the initial concentration. From its logarithmic transformation, the fall of the ICG concentration is characterized by a straight line with a negative slope that represents a percentage change in concentration with time [11,12].

Despite being a highly reliable test, the determination of the plasma disappearance of indocyanine green prior to resection could be considered invalid. This is because the resections performed in the surgical procedure do not always correspond to those previously proposed for the calculation of liver reserve. During resection, segments with different degrees of functionality could be eliminated due to the underlying pathology, making it difficult to achieve a good estimation before surgery. The completion of this determination after the resection could contribute more reliable results and provide more information in relation to the liver reserve.

By using the plasma elimination of indocyanine green on the first day of the postoperative period, it is possible to know the exact value of the hepatic function in the most realistic conditions and in a personalized way. In this way, the real function of the remaining liver will be obtained, allowing the estimation of the risk of developing hepatic insufficiency. The characteristics of the test could produce reliable personalized results earlier than the current “gold standard” (50:50 criteria on postoperative day five).

## 2. Materials and Methods

It was hypothesized that the determination of the hepatic reserve by measuring the plasma elimination of indocyanine green—after the hepatic parenchymatous resection—could provide an earlier and more exact knowledge of the hepatic reserve. The objective of the study was to determine if the ICG-PDR (LiMON^®^, PULSION Medical Systems, Munich, Germany) at 24 h after surgery correlates with the incidence of liver failure on the 5th postoperative day after liver resection in the Hepatobiliopancreatic Surgery Unit of the General University Hospital of Ciudad Real.

A prospective observational post-authorization study (EPA-SP) was conducted. The study was approved by the Ethics and Clinical Research Committee of the General University Hospital of Ciudad Real.

Patients scheduled for liver surgery, who agreed to be included in the study, and who signed the informed consent providing information about the aims of the study and its possible complications, were consecutively included in the study for a period of two years. All patients listed for liver resection who fulfilled the inclusion criteria and provided informed consent were enrolled in the study, irrespective of the underlying pathology prompting the surgical indication. The inclusion and exclusion criteria are shown in Table 1.

Before the operation, the hepatic reserve was assessed with the LiMON^®^ monitor (PULSION Medical Systems, Munich, Germany) by means of the determination of the ICG-PDR. The ICG-PDR dose used was 0.25 mg/kg. The monitor has a calculator that evaluates the amount and correct dilution of ICG based on the patient’s weight and height, dosage, and concentration of the vial contents. The monitor starts calibration and prompts for injection. The curve detected message is displayed and the measurement starts. Once this has been completed, the result is displayed in a table. For the assessment of hepatic flow and hepatic function, the plasma concentration of indocyanine green is measured by spectrophotometry at the time of injection and after 15 min.

After liver resection surgery, the patient was admitted to Post-op. Twenty-four hours after the operation, the hepatic reserve was assessed using the same technique. On the fifth postoperative day, either in postoperative phase if the patient continues to be monitored, or on the hospital ward if discharged, an analysis was taken, including blood count, coagulation, biochemistry (including liver function), total bilirubin and prothrombin time.

The EPIDAT 3.1^®^ software was used to calculate the sample size. We accepted a type I error risk (α) of 5% and a type II error (β) risk of 20, in bilateral contrast and with an expected proportion of tracking losses of 0.1. A population of *n* = 44 was required.

SPSS 15.0^®^ (Statistical Package for the Social Sciences) was used for statistical analysis. Pearson correlation coefficients were made between post-hepatectomy PDR and the classic parameters of postoperative liver failure (bilirubin and prothrombin time). In the statistical analysis, *p* < 0.05 was considered to reject the null hypothesis, deducing that the observed difference is significant, with an error probability of less than 5% (type I error). Analyses of ROC (receiver operating characteristic) curves were performed to establish the most optimal threshold for discrimination of hepatic insufficiency with the greatest possible sensitivity and specificity. In order to determine the differences between the sample means of the different tests, Student’s t test was carried out. The determination coefficient R2 was calculated to predict future results and to estimate the proportion of variation in results that can be explained by the model.

## 3. Results

The results were based on 44 patients of the General University Hospital of Ciudad Real. They were included in the surgical waiting list of the hospital’s Service of Hepatobiliary Surgery and agreed to be included in the study. Patients were included prospectively and consecutively in the study.

The demographic and analytical values of the patients are listed in Table 2 (preoperative values), Table 3 (intraoperative values) and Table 4 (postoperative values). In the postoperative period, the patients had stays of 3 to 72 days (14.51 ± 13.49 days). They presented a preoperative PDR from 5.2 to 46.4%/min (21.17 ± 7.05%/min) and a preoperative R15 from 0.1 to 41%/min (7.04 ± 8.61%/min). Postoperative PDR ranged from 2.9 to 29.8%/min (17.75 ± 7.41%/min) and postoperative R15 from 1.4 to 74.7%/min (13.5 ± 17.63%/min). On the fifth postoperative day, the following results were obtained: bilirubin, from 0.3 to 11.9 mg/dL (2.1 ± 2.84 mg/dL); prothrombin time, from 9.9 to 27.84 s (13.41 ± 3.86 s); GOT (glutamic-oxaloacetic transaminase), from 12 to 5124 IU/L (198.54 ± 833.55 IU/L) and GPT (glutamic-pyruvic transaminase), from 13 to 1426 IU/L (165.24 ± 228.77 IU/L).

In terms of mortality, 36 patients (81.8%) had survived at six months. Regarding postoperative complications, ten patients (22.7%) had renal failure (five patients (11.36%) were suffering from renal failure before de intervention), seven patients (15.9%) had heart disease, fourteen patients (31.8%) developed respiratory failure, six patients (13.6%) had sepsis, two patients (4.54%) had liver failure on the fifth postoperative day, and two patients (4.7%) had recurrence.

**Figure 1:**
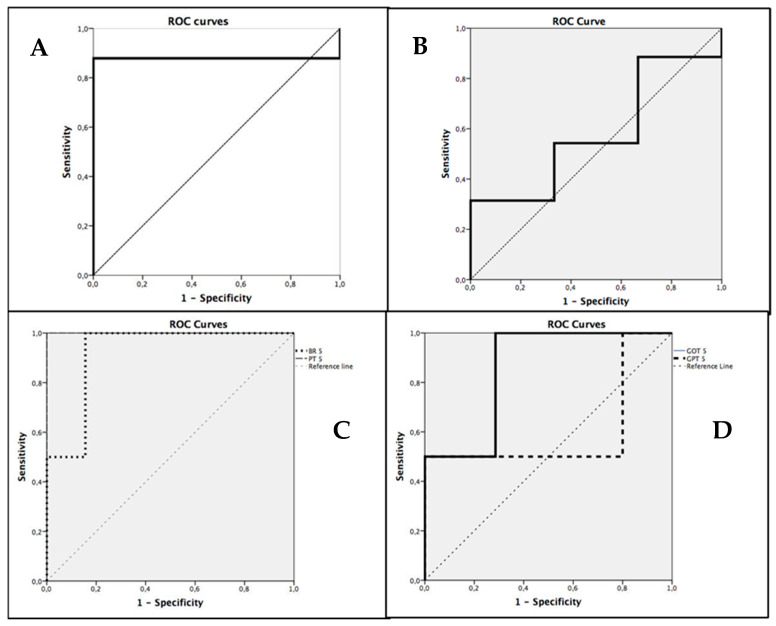
ROC Curves for PDR prediction N = 44. (**A**) ROC curve for Postoperative PDR. (**B**) ROC curve for Preoperative PDR. (**C**) ROC curves for Bilirubin and Prothrombin Time on the fifth postoperative day. (**D**) ROC curves for GOT and GPT on the fifth postoperative day.

Applying ROC curves and the area under the curve (AUC) for the evaluation of the different tests as predictors of hepatic failure, favorable data were obtained in relation to bilirubin (AUC = 0.922) and prothrombin time (AUC = 1), and for postoperative PDR (AUC = 0.879) and GOT (AUC = 0.857), but not for preoperative PDR (AUC = 0.667) or GPT (AUC = 0.6) (Figure 1).

The cut-off point with the highest sensitivity and specificity for the postoperative PDR was 7.8%/min %) (95% confidence interval ranges from 7.67%/min to 9.90%/min) (sensitivity of 87%, specificity of 50%).

In order to measure the degree of relationship between the variables, the Pearson correlation coefficients were calculated. The postoperative PDR has a good correlation with bilirubin (*p* < 0.01) and with prothrombin time (*p* = 0.001).

**Figure 2:**
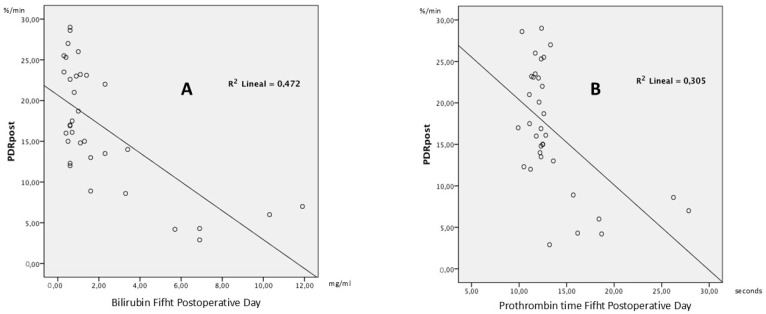
Scatter Plots of PDR and biochemical parameters. (**A**) Scatter plot of postoperative PDR versus bilirubin on postoperative day 5 (*n* = 44). (**B**) Scatter plot of postoperative PDR versus prothrombin time on postoperative day 5 (*n* = 44).

In order to test the hypothesis and determine the quality of the model for replicating the results, and the proportion of variation in results that can be explained by it, the linear R2 was calculated, defined as the square of Pearson’s correlation coefficient. This expresses the proportion of response variability explained by a regression model. In the case of postoperative PDR and bilirubin (Figure 2), 47.2% of all variability of the PDR is explained by bilirubin. In the case of postoperative PDR and prothrombin time (Figure 2), 30.5% of all variability of the PDR is explained by prothrombin time. This reflects that in 47.2% of patients, when total bilirubin falls on the fifth postoperative day, the same trend is followed by postoperative PDR. In 30.5% of patients, postoperative PDR follows the same trend as prothrombin time.

## 4. Discussion

Liver failure following liver resection is a life-threatening complication. Its incidence ranges from 4% to 18% depending on the characteristics of the patients and the extent of the resection [13,14,15,16,17], although in the most recent studies there are incidences of less than 10% in patients for whom intra- and postoperative management is optimized [18]. In our study, the frequency of liver failure on the fifth postoperative day was 4.54%, possibly because different histopathological types were included in the research.

Early detection and subsequent treatment is therefore of paramount importance. Numerous tests have been used for the detection of liver failure, and currently the 50:50 criteria is used the most for early diagnosis [19]. The test has a sensitivity of 70% and a specificity of 93% if calculated on the fifth postoperative day; but, as the authors of the research admit [19], on the fifth day the measurement may be too late. As with the others [19], our working group considers that the delay until the fifth postoperative day overly postpones the diagnosis, diminishing the options of the professionals in charge of the patient.

In our study, we found that the most frequent complication was respiratory failure in fourteen patients (31.8%), followed by renal failure in ten patients (22.7%). Seven patients (15.9%) had heart disease and six patients (13.6%) developed sepsis. In 2007, Virani et al. [20] conducted a study to measure morbidity and mortality in 14 hospitals in the USA and found a complication rate of 22.6%. Whenever these studies have been carried out, surgeries of the same complexity are compared; however, the aetiologies and the extent of the resections vary greatly in our series of patients.

The presence of such a high morbidity in our study, as compared to other studies, may be because most of our patients undergo very extensive surgery that in many cases requires manipulation of the bile duct. This triggers a systemic inflammatory response in the postoperative period, requiring mechanical ventilation and leading to renal insufficiency in the first days of the postoperative period, and this could explain these numbers. Regarding mortality, our results are more in line with the figures found in the literature. Another way of understanding these numbers is that earlier detection of liver failure may lead to earlier action. Observing low ICG-PDR values makes us more alert, and thus we optimize more and maintain these measures longer in the post-operative critical care unit.

Although ICG may have some adverse effects such as hypersensitivity reactions, nausea, hives, rash and tachycardia [21,22], in our patient series, we did not observe any adverse reactions to the drug. Therefore, we consider indocyanine green to be a safe drug, as long as the guidelines of the product data sheet are followed.

The use of indocyanine green for liver function assessment is not new. We found research from the late 1950s in which ICG was used to assess liver function in dogs [23]. In the early 1970s, human studies were initiated [11,24,25]. At the end of the 1990s, studies began to assess hepatic function prior to resection and to explore the feasibility of surgery without the risk of postoperative hepatic failure, laying the groundwork for approaches now integrated into personalized medicine. Pei Liu et al. [26], found that capillarization of hepatic sinusoids is one of the causes of liver failure. Abnormalities such as barriers between the sinusoids and hepatocytes, as well as the formation of intrahepatic shunts that affected the diffusion of albumin, were discovered. They succeeded in correlating the decrease in the extraction of indocyanine green with the decrease in the extravascular space accessible to albumin.

Lam et al. [27] selected a group of patients with hepatocellular carcinoma who were to undergo major hepatectomy (defined as a resection of three or more hepatic segments) and divided them into high-risk (ICG retention at 15 min > 14%/min) and low-risk (ICG retention at 15 min < 14%/min). They obtained similar results in both groups, considering that the retention of ICG as the sole predictor of hepatic failure, using 14% as the safety limit, is not valid. Imamura et al. [7] designed a decision tree based on three variables—ascites, total bilirubin and the ICG—and applied it to 1429 hepatectomies, of which 685 were due to hepatocellular carcinomas. They found that this decision tree is an accurate tool for estimating the amount of liver that can be removed during the surgery, although they limit its use to centers with experience in hepatobiliary surgery.

All these studies use the ICG as a preoperative tool, to try to delimit the amount of hepatic tissue that can be resected in surgery.

In our study, we found a good correlation between postoperative ICG-PDR (*p* = 0.012), bilirubin (*p* = 0.007) and prothrombin time (*p* = 0.003). These results agree with other research in which the patients who presented hepatic failure had values of ICG-PDR lower than those who did not present it [22,28].

These results might contribute to make an earlier diagnosis possible, allowing us to anticipate the gold standard test that we were currently using. The 50:50 criteria delays the diagnosis until the fifth postoperative day, whereas with the postoperative ICG-PDR, it is advanced to the first postoperative day, enhancing early risk stratification in the context of personalized medicine.

When applying an ROC curve to the preoperative PDR, the area under the curve indicates that it is not a good test to predict liver failure (AUC = 0.667); it has a good correlation with bilirubin (*p* = 0.003) but not with prothrombin time (*p* = 0.079). This may be due to the fact that prior to resection, when measuring the functionality of the liver, some areas are taken into account which may have full activity, but in others there may be a decrease in activity. The residual function of the organ after surgery will be different if the resected area had more or less activity, so the diagnosis of liver failure depends on variables (functionality and activity of the resected liver) that have not been measured. Hiroyuki Sugimoto’s working group [22] found that preoperative values of ICG elimination rate did not result in statistically significant differences between patients with liver failure and those without.

We have calculated the most sensitive and specific cut-off point for postoperative ICG-PDR in diagnosing hepatic insufficiency. In our study, a postoperative ICG-PDR of 7.8%/min provides a sensitivity of 87% and a specificity of 50% for the diagnosis of postoperative liver failure. In reviewing the literature, we have not found a value for the ICG on the first postoperative day that would allow the diagnosis of liver failure after liver resection. The limited specificity of these findings necessitates further validation through multicenter studies with larger sample sizes to ensure their generalizability and clinical applicability. In several articles there are numbers that allow the diagnosis of liver failure after a transplant. Olmedilla et al. [29] observed that a PDR value of 10.8%/min at 24 h after a liver transplant contradistinguishes the two different risk populations, with 100% sensitivity and 90.4% specificity. Levesque et al. [30] have a cut-off point for complications after liver transplantation of 12.85%/min. Vittorio Cherchi et al. [31] found that an ICG-PDR of less than 16% per minute on the first postoperative day was significantly associated with early graft dysfunction (EAD) and with mortality at three months, twelve months, and five years. Therefore, routine measurement of ICG-PDR on the first postoperative day is suggested as a useful method for predicting both short- and long-term outcomes.

Their results were higher than those obtained in our study. This may be because theirs was not the same target population, their studies were performed after liver transplant surgery and our patients underwent resective surgery. Therefore, the clinical circumstances and complications of the two groups were different, and they cannot be compared. In transplantation, surgical aggression is greater than in resection, and it may take longer for the liver to begin to perform its adequate function; hence, there is a greater need for post-operative ICG-PDR values to alert us to the potential risk of liver failure. In our work, with regard to resections, we provided a slightly lower value for alerting about the risk of liver failure in a personalized way.

These findings may inform the development of clinical guidelines or decision-support algorithms aimed at optimizing postoperative management in this patient population, contributing to more individualized care within the scope of personalized medicine.

The study includes 44 patients who underwent liver resection for a range of pathologies and was conducted at a single center, which may limit the generalizability of the findings. Only two patients (4.54%) developed liver failure. Further multicenter studies are warranted to validate and strengthen these results.

## 5. Conclusions

We therefore conclude that the gold standard for predicting early liver failure (the 50:50 criteria on the fifth postoperative day) has a good relationship with the plasma elimination rate of indocyanine green on the first postoperative day. The administration of indocyanine green in the postoperative period following hepatic resection may represent a valuable tool in the clinical management and monitoring of these patients, particularly when integrated into strategies aligned with personalized medicine.

## Figures and Tables

**Table 1:** Inclusion and Exclusion Criteria from the Study.

Inclusion Criteria	Exclusion Criteria
Patients susceptible of receiving Surgical treatment	History of Hypersensitivity to Indocyanine Green or Iodine
Liver Disease with Surgical Indication	Thyroid Pathology
Adulthood	Underage Status
Accept Inclusion	Pregnant
	Irresectable Tumors

**Table 2:** Preoperative details (*n* = 44).

	Average	Standard Deviation
Age (Years)	60.75	±13.599
Weight (kg)	80.6207	±23.61660
Height (Meter)	1.6559	±0.07258
Hemoglobin (g/dL)	12.7409	±1.65618
HCTO (%)	37.8682	±4.74816
Creatinine (mg/dL)	0.8905	±0.37130
Urea (mg/dL)	36.0909	±14.20746
PDR pre (%/min)	21.1690	±7.05527
R15 pre (%)	7.0396	±8.60916

**Table 3:** Intraoperative details.

Laparoscopic Procedures, *n* (%)		
	Laparoscopy	10 (22.7%)
	Laparotomy	34 (77.3%)
Blood Loss (mL)	Mean ± SD	403.1026 ± 810.12045
Liver segments resected, *n* (%)		
	No	2 (4%)
	1	5 (11%)
	2	15 (34%)
	3	13 (30%)
	4	6 (14%)
	5	2 (4%)
	6	1 (2%)
Number of Injuries (Metastases or Implants), *n* (%)		
	1 Injury	28 (70%)
	2 Injuries	8 (20%)
	4 Injuries	1 (2%)
	5 Injuries	1 (2%)
	8 Injuries	1 (2%)
	15 Injuries	1 (2%)

**Table 4:** Postoperative details.

Stay (Days)	Mean ± SD	14.51 ± 13.493
Hemoglobin (gr/dL)	Mean ± SD	11.2930 ± 1.43401
HCTO (%)	Mean ± SD	34.0395 ± 5.06304
PDR post (%/min)	Mean ± SD	17.7525 ± 7.41098
R15 post (%)	Mean ± SD	13.4978 ± 17.63281
BT 5 PO day (mg/dL)	Mean ± SD	2.1 ± 2.83736
TP 5 PO day (s)	Mean ± SD	13.4094 ± 3.85757
GOT 5 PO day (UI/L)	Mean ± SD	198.5405 ± 833.55156
GPT 5 PO day (UI/L)	Mean ± SD	165.2432 ± 228.76788
Histology, *n* (%)		
	Colorectal Liver Metastases	23 (52%)
	Non-Endocrine Metastases	5 (11%)
	Cholangiocarcinoma	4 (9%)
	Hepatocarcinoma	3 (7%)
	Gallbladder Cancer	3 (7%)
	Hepatic Adenoma	2 (5%)
	Focal Nodular Hyperplasia	1 (2%)
	Liver Cyst	1 (2%)
	Other	2 (5%)
Six Months Survival, *n* (%)		
	Survival	36 (81.8%)
	Mortality	8 (18.2%)
Postoperative complications, *n* (%)		
	Renal Failure	10 (22.7%)
	Heart Disease	7 (15.9%)
	Respiratory Insufficiency	14 (31.8%)
	Sepsis	6 (13.6%)
	Liver Failure	2 (4.54%)
	Recidiva	2 (4.7%)

## Data Availability

The original contributions presented in this study are included in the article. Further inquiries can be directed to the corresponding authors.

## Abbreviations

The following abbreviations are used in this manuscript:

ROC	Receiver Operating Characteristic
AUC	Area under the curve
PDR	Clearance
GOT	Glutamic-oxaloacetic transaminase
GPT	Glutamic-pyruvic transaminase
ICG	Indocyanine green
LiMON^®^	Liver Monitor
ICG-PDR	Indocyanine green clearance
EPA-SP	Prospective observational post-authorization study
SPSS	Statistical Package for the Social Sciences
R15	ICG retention at 15 min
